# High incidence of catheter-associated urinary tract infections and related antibiotic resistance in two hospitals of different geographic regions of Sierra Leone: a prospective cohort study

**DOI:** 10.1186/s13104-023-06591-w

**Published:** 2023-10-31

**Authors:** Sulaiman Lakoh, Le Yi, James B.W. Russell, Juling Zhang, Stephen Sevalie, Yongkun Zhao, Joseph Sam Kanu, Peng Liu, Sarah K. Conteh, Christine Ellen Elleanor Williams, Umu Barrie, Olukemi Adekanmbi, Darlinda F. Jiba, Matilda N. Kamara, Daniel Sesay, Gibrilla F. Deen, Joseph Chukwudi Okeibunor, George A. Yendewa, Xuejun Guo, Emmanuel Firima

**Affiliations:** 1 College of Medicine and Allied Health Sciences, University of Sierra Leone, Freetown, Sierra Leone; 2 Ministry of Health and Sanitation, Government of Sierra Leone, Freetown, Sierra Leone; 3 Sustainable Health Systems Sierra Leone, Freetown, Sierra Leone; 4 Infectious Disease Research Network, Freetown, Sierra Leone; 5 Tropical Infectious Disease Prevention and Control Center, Freetown, Sierra Leone; 6 Department of Clinical Laboratory, the Fifth Medical Center of Chinese PLA General Hospital, Beijing, 100039 China; 7 34 Military Hospital, Freetown, Sierra Leone; 8 Department of Emergency Medicine, the Fifth Medical Center of Chinese PLA General Hospital, Beijing, 100039 China; 9 Department of Medicine, College of Medicine, University of Ibadan, Ibadan, Nigeria; 10 Department of Medicine, University College Hospital, Ibadan, Nigeria; 11 World Health Organization Regional Office for Africa, Brazzaville, Congo; 12 Department of Medicine, Case Western Reserve University School of Medicine, Cleveland, OH USA; 13 Division of Infectious Diseases and HIV Medicine, University Hospitals Cleveland Medical Center, Cleveland, OH USA; 14 Johns Hopkins Bloomberg School of Public Health, Baltimore, MD USA; 15 Clinical Research Unit, Department of Medicine, Swiss Tropical and Public Health Institute, Basel, Switzerland; 16 University of Basel, Basel, Switzerland; 17 SolidarMed, Maseru, Lesotho; 18 Centre for Multidisciplinary Research and Innovation, Abuja, Nigeria

**Keywords:** Multidrug resistance organisms (MRO), Extended spectrum β-lactamase (ESBL)-producing *Enterobacteriaceae*, *Catheter-associated urinary tract Infections (CAUTI)*, WHO priority pathogens, Carbapenem resistance *Enterobacteriaceae (CRE)*

## Abstract

**Objective:**

Catheter-associated urinary tract infections (CAUTI) are common worldwide, but due to limited resources, its actual burden in low-income countries is unknown. Currently, there are gaps in knowledge about CAUTI due to lack of surveillance activities in Sierra Leone. In this prospective cohort study, we aimed to determine the incidence of CAUTI and associated antibiotic resistance in two tertiary hospitals in different regions of Sierra Leone.

**Results:**

The mean age of the 459 recruited patients was 48.8 years. The majority were females (236, 51.3%). Amongst the 196 (42.6%) catheterized patients, 29 (14.8%) developed CAUTI. Bacterial growth was reported in 32 (84%) patients. *Escherichia coli* (14, 23.7%), *Klebsiella pneumoniae* (10, 17.0%), and *Klebsiella oxytoca* (8, 13.6%) were the most common isolates. Most isolates were ESBL-producing *Enterobacteriaceae* (33, 56%) and WHO Priority 1 (Critical) pathogens (38, 71%). Resistance of *K. pneumoniae, K. oxytoca, E. coli*, and *Proteus mirabilis* was higher with the third-generation cephalosporins and penicillins but lower with carbapenems, piperacillin-tazobactam and amikacin. To reduce the high incidence of CAUTI and multi-drug resistance organisms, urgent action is needed to strengthen the microbiology diagnostic services and develop and implement catheter bundles that provide clear guidance for catheter insertion, care and removal.

## Introduction

Catheter-associated urinary tract infections (CAUTI) are common worldwide, but due to limited resources, their actual burden in low-income countries is unknown [1]. However, global estimates report that low-income countries have a higher CAUTI burden than high-income countries, with cumulative incidence densities of 8.8/1000 catheter days and 4.1/1000 catheter days, respectively [1]. In a study conducted on healthcare-associated infections (HAIs) in Ethiopia, CAUTI was the most commonly detected HAI [2].

Urinary catheters are critical in routine health care delivery, but can lead to CAUTI if not handled properly. There are several adverse events associated with CAUTI, including prolonged hospital stay, worsening in-hospital mortality, and increased healthcare costs [3–7]. Furthermore, many of the bacteria causes of CAUTI are multidrug-resistant pathogens, including extended-spectrum beta-lactamase (ESBL) producing *Enterobacteriaceae* [8, 9]. Despite these challenges, systematic support for the routine diagnosis of CAUTI and related antibiotic resistance is limited by the lack of adequate surveillance activities in some African countries [10].

It is in this context that the World Health Organization (WHO) developed a global initiative to support low- and middle-income countries in strengthening their surveillance structures for CAUTI and other HAIs [11]. However, the financial and human resources challenges hinder efforts to provide a safe hospital environment for populations in low-income countries [12].

Sierra Leone, a low-income country in West Africa, has been affected by major public health emergencies, including the largest Ebola outbreak to date [13]. Before the 2014–2016 Ebola outbreak, the country had no system to prevent HAIs [14]. Consequently, the government developed a policy to guide the implementation of infection prevention and control (IPC) practices, including the surveillance and prevention of HAIs [14]. However, this development has not brought significant changes to the surveillance of HAIs in the country. Although the Ministry of Health and its partners have begun surveillance of surgical site infections in some hospitals, Sierra Leone does not have a CAUTI surveillance system. Currently, there are gaps in knowledge about CAUTI due to limited surveillance activities in the country. At the time of writing, no studies have been published on the incidence of CAUTI in Sierra Leone. This situation, and the fact that our previous work has shown deep-rooted challenges in the implementation of IPC practices in Sierra Leone prompted us to pilot a surveillance program for CAUTI in two hospitals situated in two geographic regions of Sierra Leone [15–17]. In this study, we aimed to determine the incidence of CAUTI and associated antibiotic resistance in order to inform preventive interventions.

## Methods

### Study design

The study utilized a prospective cohort design involving primary data collection.

### Study settings

Sierra Leone is divided into five geographic regions, including the Northern province and the Western Area. The Western Area is the most densely populated region of Sierra Leone with a population of about 1.5 million [18].

The public health system is divided into primary, secondary and tertiary levels of care. The national and regional hospitals provide tertiary care. There are 25 public hospitals in Sierra Leone of which 9 provide tertiary services. The study was conducted in two of the tertiary/regional hospitals (Makeni Government Hospital: MGH and the 34 Military Hospital: MH) in different geographic regions of Sierra Leone as both are likely representative of many tertiary hospitals in sub-Saharan Africa. Both hospitals serve large populations in their catchment areas. While MH is located in Freetown, MGH is about 170 km from the capital with a catchment population of 606,544 (approximately 8.6% of the Sierra Leonean population). Both hospitals have similar infrastructure with bed capacities of 207 (regional) and 187 (capital city). They both provide medical services [18, 19].

### Study population and sampling technique

All consenting patients aged 18 years or older admitted on the medical wards of the two hospitals were enrolled in the study between March and October 2021. A sample size of 459 patients was reached over a 24-week sampling period.

A team, comprised of trained nurses on the detection of HAIs collected the required information. Patients were recruited in medical wards or intensive care units (ICU) at admission and followed until the end of admission. Sociodemographic variables, clinical parameters and information on urinary catheterization were recorded at baseline. After the baseline assessment, the patients were then monitored for features suggestive of CAUTI.

In cases of suspected CAUTI, we aseptically collect urine for urinalysis, culture, and sensitivity to diagnose CAUTI. We define CAUTI as ‘the presence of at least one of the following signs or symptoms: fever (> 38.0°C), suprapubic pain or tenderness, costovertebral angle pain or tenderness, dysuria, and urinary frequency in a patient that had an indwelling urinary catheter that had been in place for more than 2 consecutive days in an inpatient location and the presence of a positive dipstick for leukocyte esterase and/or nitrite and microorganisms seen on Gram’s stain of unspun urine and a positive urine culture of ≥ 10^5^ colony-forming units/ml with no more than two bacteria species’ [20, 21].

### Laboratory procedure

Urine samples were streaked onto the chromogenic agar plate and incubated aerobically at 37 °C for 18 to 24 h. A single bacterial colony was picked up where there is growth and then streaked onto a Brain Heart Infusion agar plate for Gram stain. Bacteria solutions were prepared to 0.5–0.63 McFarland turbidity standards in polystyrene tubes (bioMérieux, France) using a DensiCHEK Plus turbidimeter (bioMérieux, France). A suspension of each isolate was loaded onto the VITEK 2 compact system and incubated overnight at 37 °C to detect antibiotic susceptibility. *Escherichia coli*, *Klebsiella* spp., *Serratia ficario* and *Proteus mirabilis* were tested for susceptibility to cephalosporins alone and in combination with clavulanic acid. A reduction in the proportion of bacterial growths in wells containing both cephalosporin and clavulanic acid compared with wells containing cephalosporin alone was considered indicative of extended-spectrum β-lactamase (ESBL) production. Carbapenemase production was determined by analysis of carbapenem susceptibility results. Isolates were defined as susceptible, intermediate, or resistant based on the minimum inhibitory concentrations of imipenem and meropenem in the Clinical Laboratory Standard Institute (CLSI) breakpoints updated in January 2020 [22]. All culture and resistance test results are sent to research teams and service providers within 24 h.

### Data management and analysis

All the data were recorded electronically on a password protected Epicollect software platform accessible only to the study team and exported to excel format. Data was clean, coded and analyzed using the Statistical Package for Social Sciences version 21.0 (Armonk, NY: IBM Corp). Descriptive statistics such as frequencies and percentages were used to present demographic, clinical characteristics of study participants and antibiotic resistance profile.

## Results

### Demographic characteristics of study participants

Most of the study participants were treated in MH (245, 53.4%). There were 236 (51.3%) female patients. The mean age was 48.8 years (SD, 18.0) (Table 1).

**Table 1:** Demographic characteristics of study participants

Parameter	Total N(%)	MH n(%)	MGH n(%)
**Overall total**	459(100)	245(53.4)	214(46.6)
**Sex**			
Female	236(51.4)	122(49.8)	114(53.3)
Male	223(48.6)	123(50.2)	100(46.7)
**Age(yrs)**			
Mean (SD)	48.8(18.0)	53.3(15.9)	43.7(18.8)
**Marital status**			
Single	102(22.2)	47(19.2)	55(25.7)
Married	291(63.4)	159(64.9)	132(61.7)
Separated/divorced/widowed	66(14.4)	39(15.9)	27(12.6)
**Education**			
None	192(41.8)	57(23.3)	135(63.1)
Primary	29(6.3)	18(7.3)	11(5.1)
Secondary	159(34.6)	111(45.3)	48(22.4)
Tertiary	79(17.2)	59(24.1)	20(9.4)
**Occupation**			
None	74(16.1)	37(15.1)	37(17.3)
Student	44(9.6)	19(7.8)	25(11.7)
Informal sector	213(46.4)	85(34.7)	128(59.8)
Formal sector	82(17.9)	61(24.9)	21(9.8)
Retired	46(10.0)	43(17.5)	3(1.4)
**Social history**			
Never smoked, never taken alcohol	372(81.1)	203(82.9)	169(79.0)
Only smokes	23(5.0)	13(5.3)	10(4.7)
Only takes alcohol	19(4.1)	9(3.7)	10(4.7)
Smokes and drinks alcohol	45(9.8)	20(8.2)	25(11.7)

*MH = 34 Military hospital; MGH = Makeni government hospital*

### Clinical characteristics, catheterization and development of CAUTI

Twenty-seven (8.1%) patients were admitted to ICU. Overall, 96(21%) patients had been admitted in a hospital in the preceding 3 months. Fever occurred in 24 (12.2%) patients.

One hundred and ninety-six (42.7%) patients were either admitted with a urine catheter or had one placed during the course of their admission. Although the reason for catheter placement was not stated in 21 (11%) cases, the commonest (95, 48.5%) reason for urinary catheter placement was urinary incontinence. Urinary catheter was in situ for a mean (SD) number of 4 (2) days, and was in place for over 3 days in 87(44.4%) patients. Of 196 catheterized patients, 38 (19.4%) had suspected CAUTI, with fever being the most common feature (Table 2). On urinalysis, 36(18.6%) patients either had a positive leucocyte esterase and/or nitrite in their urine (Table 2).

**Table 2:** Clinical characteristics and findings on the urinalysis of the study participants

Parameter	Total N(%)	MH n(%)	MGH n(%)
**Ward**			
Medical	422(91.9)	244(99.6)	178(83.2)
ICU	37(8.1)	1(0.4)	36(16.8)
**Previous hospital admission in last three months**			
No	362(79.0)	214(87.3)	148(69.5)
Yes	96(21.0)	31(12.7)	65(30.5)
Symptoms*Φ			
Fever	24(12.2)	10(8.5)	14(18.0)
Suprapubic pain	21(10.7)	13(11.0)	8(10.2)
Urinary frequency	22(11.2)	11(9.3)	11(14.1)
Dysuria	15(7.6)	10(8.4)	5(6.4)
Loin pain	5(2.6)	4(3.4)	1(1.3)
**Presence of urinary catheter**			
No	263(57.3)	127(51.8)	136(63.5)
Yes	196(42.7)	118(48.2)	78(36.5)
**Reasons for catheterization** ^*^			
Urinary retention	41(20.9)	23(19.5)	18(23.1)
Urinary incontinence	95(48.5)	87(73.7)	8(10.3)
Ambulatory dysfunction	39(19.9)	0	39(50)
Reason not stated	21(10.7)	8(6.8)	13(16.7)
**Suspected CAUTI** ^*^	38(19.4)	24(20.3)	14(17.9)
**CAUTI** ^*^	29(14.8)	19 (16.1)	10(12.8)
**Findings on urinalysis**			
Leucococyte estrase	26(13.3)	15 (75.0)	11 (84.6)
Nitrite	3 (1.5)	1(5.0)	2(15.4)
Leucocyte estrase plus nitrite	7(3.6)	4(20.0)	0(0.0)

*Denominator is number in whom urinary catheter was present (i.e. 196). ΦMultiple answers

*MH = 34 Military Hospital MGH = Makeni Government Hospital CAUTI = catheter-associated urinary tract infection*

### Bacterial isolates in patients with CAUTI

A significant bacterial growth of ≥10^5^ colony-forming units/ml was reported in 32 patients with suspected CAUTI, giving a bacterial growth rate of 84%; 71% (10/14) for MGH and 92% (22/24) for MH. Among the 32 patients with a significant bacteriuria, 3 had more than two species of bacteria. Therefore, the CAUTI incidence is 14.8%.

A total of 59 bacterial isolates were reported for MH (38, 64.4%) and MGH (21, 35.6%). *Escherichia coli* (14, 23.7%), *Klebsiella pneumoniae* (10, 17.0%), and *Klebsiella oxytoca* (8, 13.6%) were the most common urinary bacteria isolates from both hospitals. *Enterococcus spp. (4, 10.5%), Coagulase Negative Staphylococcus spp. (4, 10.5%) and Acinetobacter baumannii complex (2, 3.4%)* were only isolated from MH. On the other hand, a single isolate of *Proteus mirabilis, Bordetella henzii* and *Methylobacterium spp.* were reported only in MGH (Table 3).

**Table 3:** Distribution and rank order of bacterial isolates from the urine of patients with CAUTI

Bacterial isolate	Rank order	Total no.		MH		MGH	
		N = 59	%	N = 38	%	N = 21	%
*Escherichia coli*	1	14	23.7	11	29.0	3	14.3
*Klebsiella pneumoniae*	2	10	17.0	7	18.4	3	14.3
*Klebsiella oxytoca*	3	8	13.6	6	15.8	2	9.5
*Pseudomonas aeruginosa*	4	5	8.5	4	10.5	1	4.8
*Rhizobium radiobacter*	4	5	8.5	2	5.3	3	14.3
*Coagulase Negative Staphylococcus spp. (haemolyticus = 3 and sciuri = 1)*	5	4	6.8	4	10.5	0	0.0
*Enterococcus spp. (faecalis = 2, faecium = 1, casseliflavus = 1)*	5	4	6.8	4	10.5	0	0.0
*Acinetobacter baumannii complex*	6	2	3.4	0	0.0	0	0.0
*Bockholderia cepacia complex*	6	2	3.4	0	0.0	2	9.5
*Sphingomonas paucimobilis*	7	1	1.7	1	2.6	0	0.0
*Serratia ficario*	7	1	1.7	0	0.0	1	4.8
*Bordetella henzii*	7	1	1.7	0	0.0	1	4.8
*Proteus mirabilis*	7	1	1.7	0	0.0	1	4.8
*Methylobacterium spp.*	7	1	1.7	0	0.0	1	4.8

### Patterns of antibiotic resistance of urine isolates

**Fig. 1:**
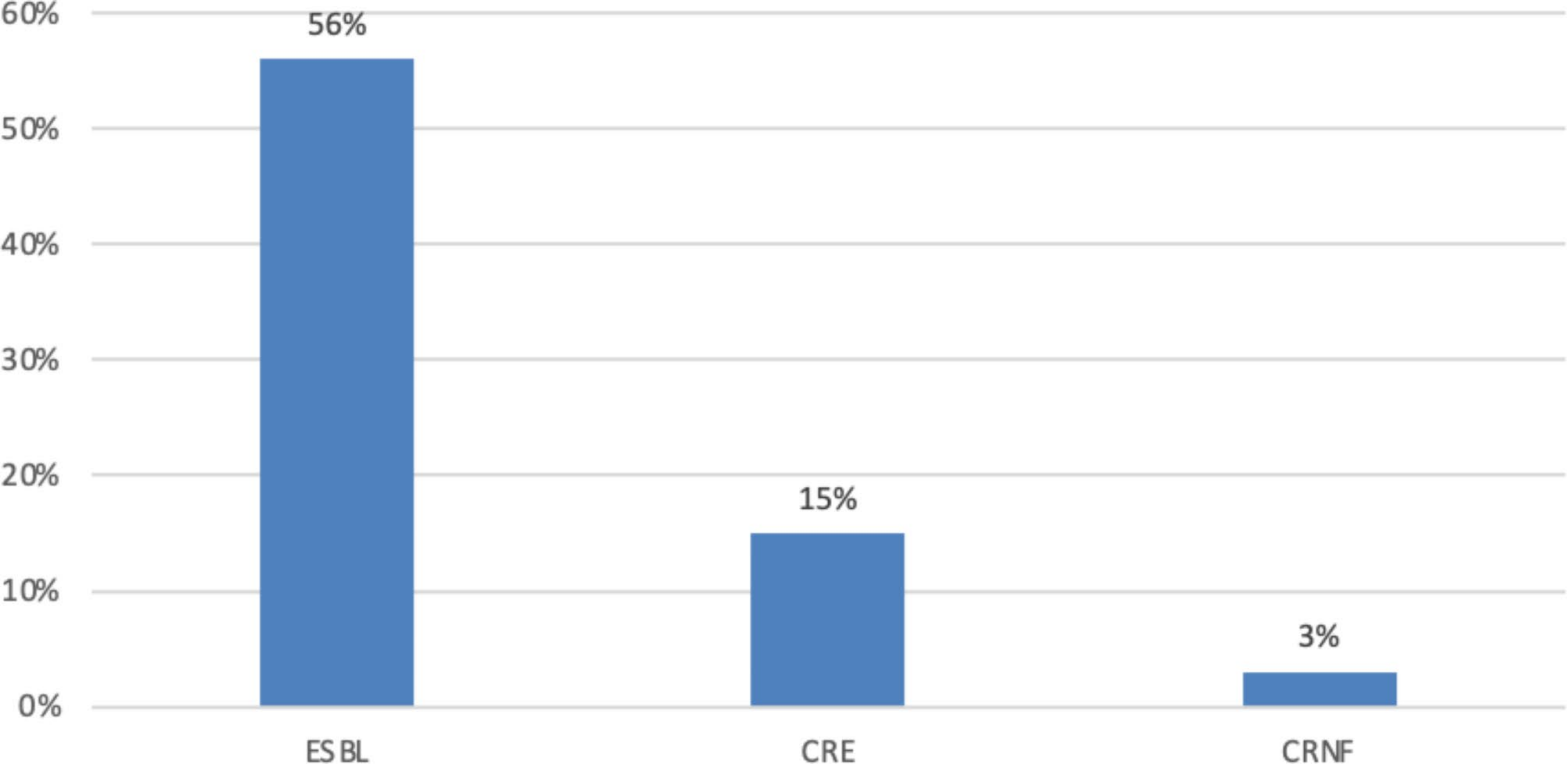
Patterns of multi-drug resistance of bacteria isolates from patients with CAUTI. *ESBL = Extended spectrum β-lactamase producing Enterobacteriaceae CRE = Carbapenem Resistance Enterobacteriaceae CRNF = Carbapenem Resistance Non-lactose fermenters*

Among the multidrug resistance bacteria, 33 (56%) were ESBL-producing *Enterobacteriaceae* and 5 (15%) were carbapenem-resistant *Enterobacteriaceae* (Fig. 1). There were 38 (71%) isolates in the WHO Priority 1 (Critical) Pathogens List, but none of the isolates was in the Priority Pathogens Lists 2 and 3.

Resistance to the third-generation cephalosporins, penicillins, aztreonam, tetracyclines, quinolones, and co-trimoxazole was high in *K. pneumoniae, K. oxytoca, E. coli*, and *Proteus mirabilis*. In contrast, meropenem, imipenem, piperacillin-tazobactam, amikacin and nitrofurantoin showed relatively low resistance to most isolates (Table 4).

**Table 4:** Antibiotic resistance profile of Gram-negative bacteria isolates

Antibiotics	Resistance rate (%) ®							
	*E. coli* *N = 14*	* K. pneumoniae* *N = 10*	* S. paucimobilis* N = 1	*P. aeruginosa* N = 5	* A.* *baumanii* *N = 2*	* K. oxytoca N = 8*	*P. mirabilis N = 1*	*B. cepacia* *N = 2*
Imipenem	7.10	10.0	0.0	0.0	0.0	0.0	100.0	-
Meropenem	7.10	20.0	0.0	0.0	-	0.0	100.0	-
Ampicillin	92.9	-	100.0	-	100.0	-	-	-
Ampicillin-sulbactam	71.4	100.0	100.0	-	50.0	35.0	-	-
Ciprofloxacin	85.7	100.0	100.0	20.0	100.0	85.7	100.0	-
Amikacin	7.1	100.0	100.0	0.0	100.0	7.1	0.0	-
Gentamicin	100.0	100.0	100.0	40.0	100.0	71.4	100.0	-
Tobramycin	78.6	100.0	100.0	40.0	100.0	78.6	100.0	-
Aztreonam	100.0	100.0	100.0	-	-	100.0	-	-
Co-trimoxazole	50.0	100.0	100.0	-	0.0	50.0	-	-
Nitrofurantoin	10.0	50.0	100.0	-	100.0	37.5	-	-
Ceftriaxone	100.0	100.0	100.0	-	100.0	100.0	100	-
Cefuroxime	100.0	100.0	100.0	-	100.0	100.0	-	-
Cefotetan	100.0	30.0	100.0	-	100.0	21.4	100.0	-
Cefazolin	100.0	100.0	100.0	-	100.0	100.0	-	-
Ceftazidime	85.7	90.0	100.0	20.0	100.0	50.0	100.0	-
Cefepime	78.6	70.0	100.0	20.0	100.0	78.6	100.0	-
Piperacillin/tazobactam	28.6	30.0	100.0	00.0	0.0	35.7	100.0	-

## Discussion

The CAUTI incidence reported in this study is 14.8%, which is almost similar to the CAUTI incidence reported in Ethiopia (16.9%), Uganda (15.3%), and Sudan (16.4%) [23–25]. However, the incidence of CAUTI reported in our study is much higher than reported in high-income countries such as the United States (1.4%), Australia (0.9%), and Italy (6.2%) [26–28]. These findings are expected, considering the fact that many hospitals in sub-Saharan have similar challenges in providing financial and human resources to support IPC and antimicrobial stewardship (AMS) activities and reinforce the fact that regardless of where patients receive care in sub-Saharan, they may have a similar risk of developing CAUTI. To reduce the high incidence of CAUTI, international guidelines and global initiatives have been developed to prevent unnecessary catheterization, but many are not readily available or implemented in low-income countries such as Sierra Leone [29]. This may account for the high rates of CAUTI in our study and other studies in sub-Saharan Africa. Therefore, sub-Saharan African countries need a coordinated approach to prevent CAUTI and ensure the safety of their populations.

In 2017, WHO published a list of priority pathogens for antibiotic resistance, which aims to align antibiotic resistance with research and development priorities. Twelve multi-drug-resistant organisms (MROs) are classified as ‘critical’, ‘high’ and ‘medium’ priority pathogens based on the severity of antibiotic resistance [30]. ESBL producing *Enterobacteriaceae* and carbapenem-resistant *Enterobacteriaceae* are examples of multi-drug resistant bacteria in the ‘critical’ group of priority pathogens [30]. Unlike a bibliometric review of data in Bahrain, which reported mostly ‘high’ priority pathogens, 71% of the isolates in this study are ‘critical’ priority pathogens [31]. The misuse and over use of the third-generation cephalosporins especially ceftriaxone, and the challenges of hand hygiene practices in these hospitals may explain this level of critical priority pathogens [15, 16, 32–34]. Unfortunately, in low-income countries like Sierra Leone, most of the antibiotics available are sold or dispensed over the counter without a prescription. Therefore, there is an urgent need for public education and enforcement of regulations to protect these essential commodities. None of the isolates are in the high or medium priority pathogen list, but this is expected as the study focused mainly on CAUTI and many of the MRO in these groups are not typical urinary pathogens [30].

Among the isolates in the critical pathogen group, we identified a high burden of ESBL *Enterobacteriaceae* from the urine of patients with CAUTI. The high burden of ESBL *Enterobacteriaceae* is a persistent problem, as previous studies in Sierra Leone have reported a similar ESBL burden in patients with surgical site infections [35, 36]. As alluded to earlier, the inappropriate antibiotic use and the challenges of hand hygiene compliance and implementation reported in some facilities in Sierra Leone may underlie the spread of ESBL *Enterobacteriaceae* in our healthcare settings [15, 16, 32–34]. To address the long-term threats associated with the persistent burden of ESBL *Enterobacteriaceae* in our hospitals, we recommend the integration of surveillance of MRO to routine clinical care and strengthen IPC and AMS interventions [37].

In the rank order of bacteria isolated from this study, *E. coli, K. pneumoniae* and *K. oxytoca* ranked first, second and third, respectively. This is not unique to this study as studies in Uganda, Ethiopia and Sudan reported similar findings [23–25]. In contrast, *P. aeruginosa* or *Enterococcus* species are the predominant pathogen isolated from the urine of CAUTI patients in Italy and Thailand [28, 38]. The variations in bacteria isolated from CAUTI patients across continents may be due to differences in environment, colonizing pathogens, or IPC practices.

Similar to studies in South Korea and Nigeria, Gram-negative bacteria isolates showed high resistance rates to the commonly available ampicillin, tetracycline and trimethoprim-sulfamethoxazole, but increase susceptibility to nitrofurantoin, piperacillin-tazobactam, amikacin and carbapenems [39, 40].

Our study is not bereft of limitations. The duration of catheterization was only considered during the hospital stay prior to the diagnosis of CAUTI. The duration of catheterization before admission and after collection of urine samples were not considered. This may have underestimated the average duration of catheter use. Although the study was conducted in two different geographic settings, we cannot generalize its findings to Sierra Leone as it was conducted only at the tertiary care level. Despite this, our study has provided the first evidence on the incidence of CAUTI in Sierra Leone.

## Conclusion

In conclusion, this study reports high rates of catheterization and CAUTI in two tertiary hospitals in Sierra Leone, most of which are associated with multidrug-resistant bacteria.

We recommend urgent action to strengthen microbiological laboratory services, integrate antibiotic-resistant surveillance into routine clinical care, and establish functional antibiotic stewardship systems. In addition, healthcare facilities in Sierra Leone and similar countries across the world should develop and implement catheter bundles that provide clear guidance for catheter insertion, care and removal.

## Data Availability

The data supporting this article is available in the repository of University of Sierra Leone and will be made available on request to the corresponding authors when required.

## Abbreviations

AMR: Antimicrobial Resistance
CAUTI: Catheter-associated Urinary Tract Infection
CRE: Carbapenem resistant *Enterobacteriaceae*
ESBL: Extended-spectrum β-lactamase
HAI: Healthcare-associated infections
ICU: Intensive Care Units
IPC: Infection Prevention and Control
MRO: Multidrug-resistant organisms
WHO: World Health Organization
